# Attitudes of the public towards halal food and associated animal welfare issues in two countries with predominantly Muslim and non-Muslim populations

**DOI:** 10.1371/journal.pone.0204094

**Published:** 2018-10-31

**Authors:** Nur Syazwani Abdul Jalil, Amanda Vrinda Tawde, Sarah Zito, Michelle Sinclair, Claire Fryer, Zulkifli Idrus, Clive J. C. Phillips

**Affiliations:** 1 Centre for Animal Welfare and Ethics, School of Veterinary Sciences, The University of Queensland, Gatton, Queensland, Australia; 2 Institute of Tropical Agriculture, and Department of Animal Science, Universiti Putra Malaysia, UPM Serdang, Selangor, Malaysia; The Hague University of Applied Science, NETHERLANDS

## Abstract

Halal food is that which is permissible or lawful for Muslims to consume. Meat products must abide by a number of requirements in relation to their preparation, condition and content to be considered halal. We conducted a survey in order to assess the knowledge of, and attitudes towards, halal meat products in two contrasting countries, one with a majority non-Muslim population (Australia, respondent n = 565), where the most commonly followed religion is Christianity, and one with a majority Muslim population (Malaysia, n = 740). The most common reasons for avoiding halal food were animal welfare, religion and meat quality. Malaysians generally believed that halal processes led to improved meat quality, whereas Australians did not. The general consensus was in favour of legally controlling animal welfare during slaughter, supported by both Muslims and Christians. Malaysians were more aware of the main tenets of halal slaughter than Australians. However, some non-compulsory, incorrect practices were thought to be required practices by respondents in both countries, but especially in Australia. Muslims were more concerned about humane treatment of animals during halal slaughter. They generally believed that stunning is never allowed and that this view was acceptable, whereas people from other belief systems generally held the view that this was unacceptable. Religion and education were the most common factors associated with attitudes, beliefs and consumer habits concerning halal. Information from this study can help to improve understanding of attitudes to halal and provide insights to policy makers seeking to address animal welfare concerns.

## Introduction

There are approximately 1.6 billion Muslims worldwide [1], with Orthodox Muslims at least strictly observing halal practices in their food choices, i.e. food items that are religiously lawful to eat according to the Quran [2]. All food products are permissible under Islamic law unless specifically prohibited by the Quran or Sunna [3]. Prohibited products are termed “haram”.

The primary sources of Islamic law are the Holy Quran and Hadith; the basic principles of Islamic law therefore remain definite and unaltered. But the interpretation of these principles may change according to Ijma (a consensus of legal opinion) or Qiyas (reasoning by analogy) [3]. Despite the ubiquity of the world’s Muslim population, differences in interpretation and application of Islamic law may cause differences in understanding and interpreting the term ‘halal’ in contrasting modern cultures, particularly in relation to what is permissible during halal slaughter.

In Malaysia, the halal certifying body, Jabatan Kemajuan Islam Malaysia (JAKIM) requires that several criteria be met [4]: both the slaughterer and Muslim halal inspector must be practising Muslims who are mentally sound and past the age of puberty; the slaughtering knife or blade must be sharp and free from impurities; the trachea, oesophagus, carotid arteries and jugular veins must be severed, and death must be ensured before further processing occurs [4].

For stunning to be accepted, there are three pre-requisites. Firstly, the stunning equipment must be used under the control of a trained Muslim supervisor or slaughter man and should be periodically monitored by a competent Islamic authority or halal certification authority [5]. Secondly, the stunning must be temporary, and must neither kill [6, 7] nor cause permanent injury to the animal [5]; thus the stunning should be reversible, and only pneumatic, non-penetrative stunning is allowed by Halal Malaysia for imported meat [4]. Thirdly, equipment used to stun pigs must never be used for halal animals [5].

Australia currently plays a major role in supplying live meat animals for consumption to predominantly Muslim countries, including in SE Asia. There has been concern among the Australian public about some halal slaughter in Indonesia in regards to animal welfare [8]. In Australia itself, according to national standards, stunning or rendering animals unconscious prior to slaughter is required [9] in order to mitigate animal suffering during slaughter. Australian slaughter standards specific to halal state that reversible stunning must be used rather than the conventional irreversible stunning [9], which is taken to mean that the animal must be able to regain consciousness, if left to naturally do so. Most Australian abattoirs aim to ensure that animals are bled out and die before consciousness is regained following stunning [10]. There are a small number of abattoirs in Australia that have permission from State or Territory food authorities to conduct religious slaughter without any prior stunning. For cattle, stunning is still required but this occurs immediately after the throat is cut [10]. All other chickens and cattle that are halal slaughtered are stunned prior to slaughter [11, 10]. Sheep slaughtered in accordance with halal practices in Australia at abattoirs exempt from stunning will only be stunned if they are distressed (eg. vocalising, butting, attempting to flee) or loss of consciousness is taking too long [11, 10].

In Malaysia, food manufacturers are striving to become a hub for global marketing of halal products [2]. The country itself has a majority Muslim population (62% of the total population [2]) and halal products are purchased by both Muslim and non-Muslim residents [12]. Non-Muslims that are more likely to intend to buy halal products are more aware of halal certification, marketing promotion, and brands [2]. However, there is confusion amongst Muslims and non-Muslims alike about the slaughter process and what halal certification means [13]. For example, there are varying opinions on whether stunning always kills the animal, whether it causes pain to the animal, and whether mechanical slaughter and the thoracic stick are permissible in halal slaughter [14].

A small survey of Muslims in Malaysia, Singapore and Australia suggested that halal certification was the most important factor for Malaysian Muslims in selecting halal meat products, above price and cleanliness, whereas for Australian Muslims cleanliness was most important [15]. Price of the halal product was less important in selecting halal meat products in Australia than in Malaysia, but in both countries the oversight of the process was considered more important than price. Respondents were unaware of some aspects of the process, for example that halal products can be transported together with non-halal products.

The objective of this study was to evaluate the public understanding of halal food and associated animal welfare issues in two countries with predominantly Muslim and Christian populations, Malaysia and Australia, respectively. Malaysia has a relatively large Muslim population of 17 million [1], in contrast with Australia’s relatively small Muslim population of just under 400,000 [16]. An understanding of the level of knowledge of, and attitude towards, halal practices in countries with contrasting religious and cultural differences is likely to be helpful in the development of future educational and practical policies.

## Methods

### Participants and procedures

A quantitative questionnaire was designed for public responses in Australia and Malaysia. The study received ethical approval from the University of Queensland human ethics committee.

Questions about socio-demographic factors were included in the questionnaire to explore their influences on the public and their consumption and market preferences. Locations for convenience sampling within Australia and Malaysia were chosen to include a wide demographic spectrum. Socio-economic indices that rank Australian areas based on the relative advantage or disadvantage of inhabitants of the postcode area were used [17]. Within Australia, the survey was conducted in South East Queensland, in five city suburbs (Logan, Stones Corner, West End, Beenleigh, and Toowoomba) and one rural town (Gatton). These areas were selected in order to target a range of socio-economic demographics. Permission was granted by the relevant councils to conduct the survey on public property. In Malaysia, surveys were similarly conducted in urban and semi-rural public spaces in and around Kuala Lumpur and in Seremban outside the capital.

Participants were randomly selected and asked two screening questions: if they were at least 18 years of age, and if they were Australian or Malaysian residents in the respective countries. If these two requirements were met, the researchers briefly outlined the study and sought participant consent. Participants decided to either complete the survey by themselves on an iPad or in paper form, or if the participant preferred the researcher read out the questions and entered the data for the participant. The surveys were conducted on weekdays in Australia between December 2015 and January 2016 and for 10 days consecutively in Malaysia in January 2016.

### Questionnaire design

The questionnaire was divided into four sections:

1. Socio-demographic variables: gender, marital status, whether there were children in the household, age, highest level of education, religion, eating habits, area of residence and income
2. Self-reported Knowledge: Participants rated their understanding of Islam and halal slaughter practices. They were then asked to state which out of ten possible halal practices were compulsory or recommended: that a practising Muslim must conduct the procedure (compulsory), that a sharp knife must be used (compulsory), an Islamic prayer must be recited (compulsory), the entire head of the animal to be severed (not compulsory), the throat, oesophagus, jugular vein and carotid artery must be severed (compulsory), the person carrying out the slaughter must be sane (compulsory), the animal must be conscious (not compulsory), an animal must not be allowed to see another animal being slaughtered (not compulsory), the meat must be approved by an Imam (not compulsory), and the head should be facing Mecca (not compulsory). Practices may vary in the nature of compulsory/not compulsory, and are here specified according to the Malaysian halal certifying body, Jabatan Kemajuan Islam Malaysia (JAKIM) [4].
3. Beliefs: the participant was asked about their beliefs about animal welfare, Islam and halal products
4. Attitude towards halal product consumption: the participant was asked if they had a positive, negative or ambivalent attitude towards halal products, and on what that attitude was based. Options given included factors related to human health, animal welfare, cost, meat quality and certification.

Definitions were provided as follows:

- Halal: an Arabic word meaning permitted or lawful by Muslim law [18]
- Animal Welfare: an animal is in a good state of welfare if (as indicated by scientific evidence) it is healthy, comfortable, well nourished, safe, able to express innate behaviour, and if it is not suffering from unpleasant states such as pain, fear, and distress [19]
- Stunning: any mechanical, electrical, chemical or other procedure which causes immediate loss of consciousness which lasts until either the animal is killed or it recovers [20]
- Reversible stunning: the animal being able to recover naturally if slaughter does not take place
- Halal certification: endorsement that a product’s contents and manufacture has been approved by an appropriate religious authority as meeting the Islamic requirements relating to food [21]
- Tasmiyah: the Islamic prayer recited during the slaughter, in Arabic

### Pilot study

A pilot study to test the clarity of the questions, effectiveness of the iPad delivery and capture of data was conducted at the University of Queensland Gatton campus with three participants. Following this a help text was embedded differentiating between compulsory requirements in order to make a product halal and non-compulsory recommendations for halal products, and a simple definition of halal was also inserted into the preliminary page. The new questionnaire was tested on 10 new participants and no further changes were made.

### Data analysis

Multivariable ordinal logistic regression analyses using a logit link function were used to assess the significance of the relationships between respondent demographics (the independent variables) and the distribution of the Likert scale responses for each question (the dependent variable). For each independent variable (demographic factors, country, age group, area of residence and income), the most numerous response category was chosen as the reference category (e.g., for nationality the most numerous response category was Malaysian so this was chosen as the reference category). Similarly, binary logistic regression analyses with a logit link function were used to assess the significance of the demographic factors on the compulsory and recommended halal slaughter practices. Other variables were analysed with a general linear model, with the same factors as outlined for the logistic regressions. The statistical package Minitab was used for all analyses and probability values were considered significant at p <0.05.

## Results

### Demographics and differences between religions

A total of 1298 eligible participants answered the survey. Of these, 563 (43%) were Australian residents and 735 (56%) were Malaysian residents. A total of 662 participants (51%) were female, slightly more proportionately in Australia than Malaysia (Table 1). The most common age group was 18–25 for both Malaysian (51%) and Australian (32%) participants. The highest level of education was an undergraduate degree for both Australian (31%) and Malaysian (33%) participants. Income was evenly spread over the different categories in Australia, but tended to be less in Malaysia. In terms of religion, Malaysian participants predominantly self-identified as Muslims (80%), while Australian participants were most likely to identify as atheist or Christian (46 and 45%, respectively). In terms of eating habits, the majority of participants were meat-eaters (88 and 94% in Australia and Malaysia, respectively). The most common place of residence was suburban for Australian participants (64%) and inner city for Malaysian participants (70%).

**Table 1:** Demographics of Australian and Malaysian respondents compared with national data.

		Australia		Malaysia	
		% of survey sample	Australian national statistics, %	% of survey sample	Malaysian national statistics, %
Gender	Male	45.8 (258)	50.6	51.4 (378)	51.7
	Female	54.2 (305)	49.4	48.6 (357)	48.3
Age	18–25	31.9 (179)	13.8 (20–29)	51.3 (378)	15.8
	26–35	21.4 (120)	13.9 (30–39)	23.2 (171)	19.9
	36–45	13.5 (76)	14.2 (40–49)	12.6 (93)	13.0
	46–55	11.9 (67)	12.7 (50–59)	9.0 (66)	6.3
	56 & over	21.2 (119)	19.6 (60 +)	3.9 (29)	45.0
Annual income (k)	< $20 AUD / < RM10	24.7 (124)		61.7 (357)	Median RM 25920 (AUD 8681)
	$21- $39 AUD / RM10–29	20.9 (105)		17.3 (100)	
	$40- $59 AUD / RM30–49	17.9 (90)		13.5 (78)	
	$60- $79 AUD / RM50–79	14.5 (73)		4.5 (26)	
	> $80 AUD / > RM70	22.1 (111)		3.1 (18)	
Religion	Islam	2.9 (16)	2.2	80.3 (590)	61.3
	Christian	45.1 (246)	61.6	3.7 (27)	9.2
	None	45.6 (249)	22.3	0.7 (5)	0.7
	Buddhist	1.6 (9)	2.5	7.9 (58)	19.8
	Hindu	1.3 (7)	1.3	7.3 (54)	6.3
	Jewish	1.5 (8)	0.5	0 (0)	-
	Other	2.0 (11)	0.8	0.1 (1)	1.7
Education	No formal education	0.2 (1)		0.5 (4)	
	Primary school	3.4 (19)	27.0	1.5 (11)	
	High school	33.2 (185)	20.5	29.7 (219)	
	Certificate/diploma	21.4 (119)	7.3	32.3 (237)	
	Undergraduate degree	30.5 (170)	14.3	32.6 (240)	
	Post-graduate degree	11.3 (63)		3.5 (26)	
Eating habits	Meat-eating	87.9 (486)	94	92.6 (630)	
	Vegetarian/vegan	11.6 (64)	6	6.9 (47)	
	Other	0.5 (3)		0.4 (3)	
Area of residence	Inner city	15.7 (88)		69.8 (508)	62
	Suburban	64.4 (360)		20.7 (151)	
	Rural	19.9 (111)		9.5 (69)	38

• Australian national statistics taken from Australian National Bureau of Statistics (2015)

• Malaysian national statistics taken from National Department of Statistics Malaysia (2013–2017)

• Certificate/diploma from technical college

• Undergraduate degree from university

• Australian national statistics on eating habits taken from http://www.scribd.com/doc/26880337/APF-VVSQ

• Lesser than is represented by the symbol < and Greater than is represented by >

When respondents were asked to rate their understanding of Islam, Muslims (mean rating 4.2, n = 543) were more likely to claim greater understanding of Islam than people from any other religion (mean 2.5–3.6), with Jews intermediate (mean 3.6, n = 7) (Table 2).

**Table 2:** Significant (P < 0.05) effects of religion on understanding of, and attitudes towards, animal welfare and halal products.

Questions and responses	Muslim (Referentgroup)	Christian	None	Buddhist	Hindu	Jewish	Other
Rate your understanding of the religion of Islam (1 very low– 5 very high)	3.95	2.65**	2.51**	2.52**	2.93**	3.57	3.58
Rate your understanding of the process of halal slaughter (1 very poor– 5 very good)	3.95	2.60**	2.51**	2.57**	3.03**	3.63	3.83
Should animal welfare during slaughter be controlled by law (1 strongly disagree– 5 strongly agree)	4.04	4.05	4.18	3.48**	3.43**	3.63	4.08
Importance ratings (1 not important to 5 very important)							
Providing halal options within Australian/Malaysian societies is:	4.81	3.13**	3.49**	3.42**	3.75**	3.71	4.25*
In halal slaughter, The humane and respectful treatment of animals is:	4.61	3.82**	3.84**	4.00**	4.04*	3.00*	4.42
Other questions							
The quality of halal meat is: 1 significantly decreased to 5 significantly increased	4.64	2.79**	2.91**	3.25**	3.35**	2.88**	3.25*
Slaughtering animals that are conscious (not stunned) for religious reasons is: 1 very unacceptable to 5 perfectly acceptable	4.37	2.07**	1.93**	2.82**	2.92**	2.50*	2.00**
Knowing an animal product is halal: 1 preferentially avoid to 5 preferentially purchase	4.77	2.42**	2.60**	2.97**	3.31**	2.88**	3.25**

Notes: All are variables that had at least one level with a p-value of less than 0.05 on multivariable analysis

* indicating <0.05 and

** indicating <0.01, with Muslim as the referent group. Responses were measured from the mean Likert scale score for that group and question.

Education level was also strongly associated with claimed understanding (P = 0.01), which increased progressively from primary school (2.8, n = 27) to postgraduate degree (3.5, n = 87). When asked to rate their understanding of the process of halal slaughter, Muslims claimed greatest understanding (4.0, n = 543), then Jews, (3.8, n = 8), Hindus (3.0, n = 53), Christians (2.6, n = 251), and finally people of no religion (2.5, n = 186) (P< 0.001). Level of education was also strongly associated with claimed understanding of the process of halal slaughter (P = 0.01), which also increased progressively from primary school (2.8, n = 27) to postgraduate degree (3.5, n = 87). Respondents from rural areas (n = 165) claimed a greater understanding (3.3) than those in inner cities (3.1, n = 542) or suburbs (3.0, n = 482) (P = 0.007); vegans claimed a greater understanding (3.9, n = 15) than meat eaters (3.0, n = 1076) (P = 0.04), and older respondents (46+, n = 186) claimed greater understanding (3.3) than 18–25 years old (2.9, n = 510, P = 0.04).

When asked whether animals are ever stunned in halal slaughter, most respondents answered never (n = 567, 51%), but some chose only if it is reversible (n = 303, 28%) or always (n = 170, 15%). Most Muslims believed that it was never allowed (n = 387, 72%), and only 116 (22%) correctly believed it was only allowed if reversible, and 27 (5%) that it was always allowed. Most atheists (31, 52%) said only if reversible, whereas most Christians and Buddhists had approximately equal numbers in each of the three categories: never, if reversible and always (P < 0.001). Legal control of animal welfare during slaughter was supported by the general consensus (including Muslims and Christians), although Buddhists, Hindus and Jews were not sure (Table 2). Humane and respectful treatment of animals in halal slaughter was more important to Muslims than other religions, with Jews attributing it the least importance value. Muslims generally thought that the quality of halal meat is better than non-halal meat, whereas those other religions tended to be unsure. Similarly, Muslims generally thought that the slaughtering of animals that are conscious for religious reasons was acceptable, whereas other religiously identified respondents within the study thought it unacceptable. Muslims had a strong preference to buy halal meat, but Christians and those without a religion tended to avoid it, with others unsure. Conversely, Muslims were unsure whether paying extra money for animal products with high welfare standards was reasonable, but Christians and those without a religion were more prepared to do so.

### Differences between Australians and Malaysians

In Australia, participants were more likely to want to pay extra for animal products with high welfare standards (OR = 0.25, p<0.001) than those in Malaysia (Table 3). However, Malaysian participants more strongly believed that providing halal options was important than those in Australia (OR = 1.85, p<0.001). In contrast to Australian participants who were more likely to believe the quality of meat in halal slaughter was the same or decreased compared to non-halal meat, most Malaysian participants strongly believed that the quality was increased (OR = 3.16, p<0.001). Furthermore, while most Australian participants believed that slaughtering conscious animals, for religious reasons, was unacceptable, most Malaysians found it acceptable (OR = 4.68, p<0.001). Most Malaysian participants chose to buy halal meat while most Australian participants chose to avoid it (OR = 3.89, p<0.001).

**Table 3:** Significant (P<0.05) effects of country, Australia or Malaysia, on attitudes towards animal welfare and halal products.

Questions and responses	Australia	Malaysia	Odds ratio	95% Confidence interval	P value
Paying more money for animal products with high welfare standards	3.78	3.26	0.25	0.14–0.45	<0.001
Providing halal options within Australian/Malaysian societies	3.34	4.57	1.85	1.00–3.42	0.050
The quality of meat in halal slaughter	2.89	4.40	3.16	1.67–5.98	<0.001
Slaughtering animals that are conscious for religious reasons	2.02	4.10	4.68	2.58–8.49	<0.001
Preference to buy or avoid halal meat	2.56	4.46	3.89	2.06–7.35	<0.001

• Questions and responses–total numbers of respondents differ between exposure variables as not all respondents answered and within variables, percentages do not always sum to 100% due to rounding.

• Responses were measured from the mean Likert score for that group and variable.

• The odds ratio estimates the odds of a respondent answering higher or lower on the Likert scale for that question.

• Paying more money for animal products with high welfare standards was measured from 1 very unreasonable to 5 very reasonable.

• Providing halal options within Australian/Malaysian societies was measured from 1 not important to 5 very important.

• The quality of meat in halal slaughter was measured from 1 significantly decreased to 5 significantly increased.

• Slaughtering animals that are conscious for religious reasons—Likert Scale options were 1 from very unacceptable to 5 perfectly acceptable.

• Preference to buy or avoid halal meat—Likert Scale options were 1 from preferentially avoid to 5 preferentially purchase.

• Lesser than is represented by the symbol < and Greater than is represented by >

When asked whether various practices are compulsory or recommended for halal slaughter, there was overall agreement in both countries that a practising Muslim must conduct the procedure, an Islamic prayer must be recited, a sharp knife used and the throat, oesophagus, jugular vein and carotid artery must be severed (Table 4). These were all considered by most participants to be compulsory practices, especially by respondents from Malaysia. More of the remaining Malaysians than Australians thought that these were recommended, except the recital of an Islamic prayer, which more Australians thought was recommended.

**Table 4:** Number and % of respondents citing different practices as compulsory or recommended for halal slaughter.

Questions and response options	Compulsory		Odds ratio	95% Confidence interval	P value	Recommended		Odds ratio	95% Confidence Interval	P value
	Australia (n = 490/%)	Malaysia (n = 696/%)				Australia (n = 490/%)	Malaysia (n = 696/%)			
A practicing Muslim must conduct the procedure	300 (61.2%)	588 (84.5%)	0.20	0.13, 0.29	<0.001	130 (26.5%)	142 (20.4%)	1.74	1.18, 2.56	0.005
An Islamic prayer must be recited	314 (64.1%)	542 (77.9%)	0.47	0.33, 0.68	< 0.001	135 (27.6%)	296 (42.5%)	0.55	0.34, 0.78	0.001
A sharp knife must be used	295 (60.2%)	556 (80.0%)	0.23	0.15, 0.33	<0.001	173 (35.3%)	179 (25.7%)	1.80	1.26, 2.56	0.001
The throat, oesophagus, jugular vein and carotid artery must be severed	295 (60.2%)	491 (70.5%)	0.42	0.29, 0.59	<0.001	118 (24.1%)	105 (15.1%)	1.90	1.26, 2.85	0.002
The person carrying out the slaughter must be sane	234 (47.8%)	519 (74.6%)	0.18	0.12, 0.26	<0.001	134 (27.3%)	134 (19.3%)	2.19	1.48, 3.23	<0.001
The head of the animal should be facing Mecca	156 (31.2%)	378 (54.3%)	0.37	0.26, 0.52	<0.001	186 (38.0%)	277 (39.8%)	0.78	0.55, 1.09	0.150
The animal must be conscious during slaughter	179 (36.5%)	446 (64.1%)	0.25	0.18, 0.36	<0.001	105 (21.4%)	146 (21.0%)	1.03	0.69, 1.54	0.890
An animal must not be allowed to see another animal being slaughtered	119 (24.3%)	274 (39.4%)	0.06	0.45, 0.92	0.002	212 (43.3%)	251 (36.1%)	0.99	0.70, 1.39	0.93
The meat must be approved by an Imam	173 (35.3%)	196 (28.1%)	1.73	1.21, 2.48	0.003	172 (35.1%)	160 (23.0%)	1.50	1.04, 2.16	0.030
The entire head of the animal must be severed	96 (19.6%)	97 (13.9%)	2.12	1.35, 3.34	0.001	79 (16.1%)	87 (12.5%)	1.42	0.89, 2.26	0.140

• Questions and response options—total numbers of respondents differ between exposure variables as not all respondents answered.

• The odds ratio estimates the odds of a respondent answering higher or lower on the Likert scale for that question.

• Lesser than is represented by the symbol < and Greater than is represented by >

• The number of participants in the sample is referred to as “n”

A majority of Malaysians thought that the person carrying out the slaughter must be sane (which Australians were more likely than Malaysians to believe to be just recommended), the head of the animal should be facing Mecca and the animal must be conscious during slaughter, whereas only between one third and one half of Australians thought these to be compulsory. A substantial proportion of Malaysians (39%) thought that the animal must not be allowed to see another animal being slaughtered, more than the 24% of Australians. Approximately one third of Australians, but only a quarter of Malaysians believed that meat had to be, or it was recommended that it was, approved by an Imam. Only a small proportion believed that the entire head of the animal had to be severed, but more Australians that Malaysians believed this.

### Other demographic effects

#### Education

Participants with an undergraduate degree as their highest level of education had a higher perceived understanding of Islam and halal slaughter than school leavers and those with a certificate or diploma (Table 5). They were also more in agreement that animal welfare during slaughter should be controlled by law than participants with other education levels. They found it more reasonable to pay extra for animal products with high welfare standards and more important to provide halal options within Australian or Malaysian societies, compared to school leavers and certificate or diploma holders.

**Table 5:** Significant effects of education on understanding of, and attitudes towards, animal welfare and halal products.

Questions and responses	Undergraduate degree (Referent group)	School leavers	Certificate/ diploma	Post-graduate degree
Rate your understanding of the religion of Islam	3.36	3.11**	3.27*	3.26
Rate your understanding of the process of halal slaughter	3.38	3.15**	3.26**	3.15
Should animal welfare during slaughter be controlled by law	4.15	3.98*	3.88**	3.95*
Paying more money for animal products with high welfare standards is:	3.76	3.33**	3.31**	3.84
Providing halal options within Australian/Malaysian societies is:	4.29	3.78**	4.05**	3.88
In halal slaughter, the humane and respectful treatment of animals is:	4.40	4.05	4.17**	4.16
The quality of halal meat is:	3.81	3.67**	3.83*	3.48
Knowing an animal product is halal, I would:	3.77	3.46	3.72	3.18

• Questions and responses—all variables had at least one level with a p-value of <0.05 on multivariable analysis

* indicating <0.05 and

** indicating <0.01, with undergraduate degree as the referent group.

• Responses were measured from the mean Likert scale score for that group and variable.

• Perceived understanding of Islam was measured from 1 very low to 5 very high.

• Perceived understanding of halal slaughter was measured from 1 very low to 5 very high.

• Control of animal welfare during slaughter by law was measured from 1 strongly disagree to 5 strongly agree.

• Paying more money for animal products with high welfare standards was measured from 1 very unreasonable to 5 very reasonable.

• Providing of halal options within Australian/Malaysian societies was measured from 1 not important to 5 very important.

• The humane and respectful treatment of animals in halal slaughter was measured from 1 not important to 5 very important.

• The quality of meat in halal slaughter was measured from 1 significantly decreased to 5 significantly increased.

• Preference to buy or avoid halal meat was measured from 1 preferentially avoid to 5 preferentially purchase

Participants with an undergraduate degree as their highest level of education also more strongly believed that the humane and respectful treatment of animals in halal slaughter was important, compared to certificate or diploma participants. They were also less likely to believe that the quality of meat in halal slaughter was increased compared to certificate or diploma holders, but they were more likely to believe that the quality was increased than school leavers.

#### Gender

Male participants were more likely than females to have a higher self-rated understanding of Islam and to find it acceptable to slaughter animals that are conscious for religious reasons (Table 6). Females were more likely to agree that animal welfare during slaughter should be controlled by law.

**Table 6:** Significant effects of gender on understanding of, and attitudes towards, animal welfare and halal products.

Questions and response options	Male	Female	Odds ratio	95% Confidence interval	P value
Rate your understanding of the religion of Islam	3.36	3.13	0.69	0.54, 0.87	0.002
Should animal welfare during slaughter be controlled by law	3.92	4.10	1.35	1.05, 1.74	0.018
Slaughtering animals that are conscious (not stunned) for religious reasons is:	3.29	3.00	0.61	0.47, 0.80	<0.001

• Questions and response options—total numbers of respondents differ between exposure variables as not all respondents answered.

• Responses were measured from the mean Likert scale score for that group and question.

• The odds ratio estimates the odds of a respondent answering higher or lower on the Likert scale for that question.

• Perceived understanding of Islam was measured from 1 very low to 5 very high.

• Control of animal welfare during slaughter by law was measured from 1 strongly disagree to 5 strongly agree.

• Slaughtering animals that are conscious for religious reasons was measured from 1 very unacceptable to 5 perfectly acceptable.

#### Income

Participants with an annual income of less than RM10, 000 or $20, 000 AUD were more likely to agree that animal welfare during slaughter should be controlled by law than those with an income of more than RM70, 000 or $80, 000 AUD (Table 7). However, they were less likely to find it reasonable to pay extra money for animal products with high welfare standards than those with an annual income of RM50, 000 or $60, 000 AUD or above. Those in the lowest income bracket were more likely to believe that the humane and respectful treatment of animals in halal slaughter was important than those that earned RM10, 000–29, 000 or $20, 000–39, 000 AUD.

**Table 7:** Significant effects of income on attitudes towards, animal welfare and halal products.

Questions and responses	< RM10, 000/ $20, 000 (Comparative group)	RM10, 000–29,000 / $20, 000–39, 000	RM30, 000–49, 000 / $40, 000–59, 000	RM50, 000–69, 000 / $60, 000 – 79, 000	> RM70, 000 / $80, 000
Should animal welfare during slaughter be controlled by law	4.00	4.10	4.21	4.02	3.87**
Paying more money for animal products with high welfare standards is:	3.39	3.33	3.54	3.83*	3.97**
In halal slaughter, the humane and respectful treatment of animals is:	4.36	3.95*	4.19	4.10	4.03

• Questions and responses—all variables that had at least one level with a p-value of >0.05 on multivariable analysis

* indicating <0.05 and

** indicating <0.01 with less than RM10,000/Less than $20, 000 as the referent group.

• Responses were measured from mean Likert score for that group and variable.

• Control of animal welfare during slaughter by law was measured from 1 strongly disagree to 5 strongly agree.

• Paying more money for animal products with high welfare standards was measured from 1 very unreasonable to 5 very reasonable.

• The humane and respectful treatment of animals in halal slaughter was measured from 1 not important to 5 very important.

• Lesser than is represented by the symbol < and Greater than is represented by >

#### Age

**Fig 1:**
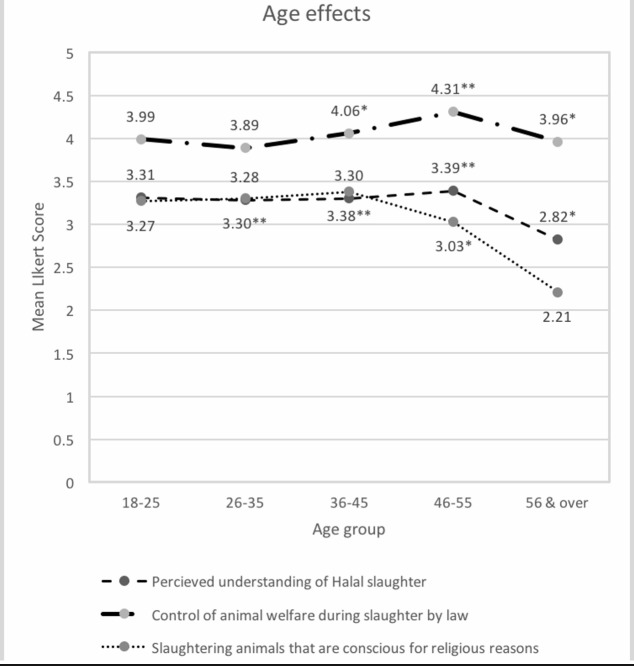
Significant effects of age on understanding of, and attitudes towards, animal welfare and halal products, with groups compared to the referent group 18–25. Variables had at least one level with a p-value of less than 0.05 on multivariable analysis, *indicating <0.05 and **indicating <0.01 with Muslim as the referent group.

Participants aged 56 and over had a lower self-rated understanding of halal than other age groups (Fig 1). Respondents aged 46–55 were more likely to agree that animal welfare during slaughter should be controlled by law than those aged 18–25 and 56 and over. Agreement with slaughtering animals that are conscious for religious reasons declined in older respondents.

#### Reasons for avoiding or purchasing halal animal products

**Fig 2:**
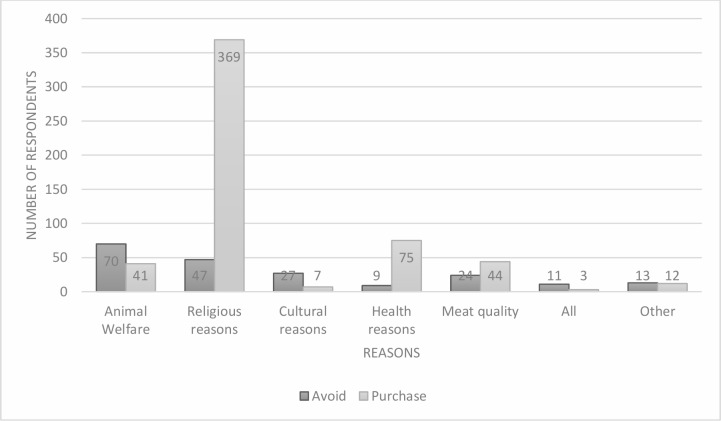
Reasons for avoiding and purchasing halal animal products.

The most popular reason for purchasing halal animal products was for religious reasons (n = 369), while the most common reason for avoiding these products was for animal welfare reasons (n = 70) (Fig 2).

## Discussion

The respondent population was broadly similar to national statistics, the primary difference being in the religious affiliation of the respondents, being predominantly Muslim for Malaysians and Atheist/Christian for Australians. As a result the differences between Muslims and Christians detected were similar to those between Malaysians and Australians. However, it must be noted that we based our geographical representation, in the case of Australia, on socioeconomic indices and were not able to do that for Malaysia. Hence we cannot be absolutely sure that the two populations were exactly comparable.

Demographic factors such as country, religion and education were significantly associated with attitudes, beliefs and consumer habits regarding halal slaughter practices. These findings are in accordance with an earlier study that measured knowledge regarding Halal in non-Muslim Malaysian residents, examining the impact of various socio-economic/demographic factors. The study found that “those who are more religious and the urban dweller seem to more likely to be aware of the advantages of Halal principles” [22]. This study also presented generally accepted principles that a social network, opinions and beliefs are circulated and shared by many of its members, thus determining attitudes, expectations and actions [23].

The present study found that participants with a lower annual income were more likely to agree that animal welfare during slaughter should be controlled by law, but less likely to find it reasonable to pay extra money for animal products with high welfare standards than those with an annual income of RM50, 000 or $60, 000 AUD or above. One possible explanation for this is that those with less disposable income would prefer laws to be imposed to improve welfare standards across all products, as they do not have the perceived luxury of additional expenditure in order to purchase a more ethical product. Koos [24] concluded that a high equivalent income enables consumers to purchase certain goods for ethical, environmental or political reasons and that “people in higher-class positions use their purchasing power as a means of political voice more often than people in lower-class positions”.

Those respondents in the lowest income bracket were also more likely to believe that the humane and respectful treatment of animals in halal slaughter was important, however other studies have found that high-income respondents had a higher level of concern regarding animal welfare, which is at odds with these results [25]. This is a complex issue with numerous socio-economic and socio-political factors.

### Knowledge gaps

The differences in overall attitudes to halal animal products may be attributed in part to the disparity in knowledge of halal slaughter techniques between Australian and Malaysian participants, with Australians possessing less understanding than Malaysians. For instance, Australian participants were more likely than Malaysians to incorrectly identify that halal meat must be approved by an Imam and that the entire head of the animal must be severed as compulsory practices in halal. The latter is contrary to the Sunnah, a verbally-transmitted record of the Prophet Mohammad’s teachings, which requires that the spinal cord should be intact in halal slaughter.

Australians were also more likely to believe that certain practices are only recommended when actually they are compulsory in halal slaughter; for example that halal slaughter must be conducted by a practising Muslim. This demonstrable lack of familiarity with Muslim practices in Australia could be explained by the much smaller proportion of Muslims in the Australian population (2%) compared to the Malaysian population (61%) [26, 15]. It is also likely that non-Muslim Malaysians possess more knowledge of halal through living in a majority Muslim country and having more exposure to Islamic practices such as halal slaughter. This is further supported by Golnaz et. al. [22] who found that non-Muslim consumers in Malaysia were aware of the existence of halal principles and methods of slaughter and the reasons for them.

Malaysian respondents may also be more familiar with halal practices regardless of their religious beliefs in part due to the state government of Malaysia taking on the role of a halal-certifying authority and standardising the processes [27]. However, they still demonstrated an adherence to a standard set of procedures, which were also agreed by Australian respondents (Table 4). This is probably due to people’s general understanding of the Muslim faith, although the drivers of such attitudes have yet to be thoroughly explored. Policy makers must understand the gaps between common perceptions and the prescribed requirements of halal slaughter in the relevant religious texts. A clear and open debate to clarify what is required is long overdue. The present study particularly demonstrated a lack of knowledge amongst Malaysian Muslims in regards to permissible stunning. This supports previous findings that Muslim respondents were unaware of some aspects of the halal process [15].

This lack of knowledge could result in opportunity for confusion and increased possibly of being misled. A study into the perspectives of halal restaurant operators in Malaysia highlighted individual frustrations at the ease with which Muslim customers could still be misled, with one operator stating “I believe that halal certification has no meanings because mostly the non-Muslims will desperately put their own halal signage at their eating premise to attract the Muslim customers; this is because their foods are not entirely and genuinely halal” [28]

Religion appeared to be the basis of acceptance of animal slaughter without the use of stunning, which was linked to the acceptance of halal food. Muslims may believe that the bleed time of stunned animals is adversely affected by stunning, although neither head only electrical nor captive bolt stunning affects this [29, 30]. A low frequency head to back stun has been shown to reduce bleed out in goats, with consequent reduction in microbiological quality of the meat [31]. Stunning accelerates loss of consciousness, with benefits for animal welfare [32]. However, some Muslim consumers also fear that the stunning process may kill animals prior to the throat being cut, which would make the meat unacceptable for consumption [15].

While Muslims thought that the slaughtering of animals that are conscious for religious reasons was acceptable, all other religiously identified respondents within the study thought it unacceptable. This is a point of interest in regards to the small number of Jewish participants, as it suggests a possible lack of knowledge of the process of Kosher slaughter, which does not permit pre-slaughter stunning [33].

Muslim consumers may prefer transacting with Muslim butchers because they are individuals of known reputation with similar moral and religious obligations, as this may confer confidence that animals are slaughtered and the meat is prepared with respect to religious rituals [15].

There was also a major difference between Malaysian and Australian participants regarding opinions on meat quality in halal. While most Australians believed that the quality of meat was unaffected or decreased as a result of halal slaughter, the majority of Malaysians were of the opinion that the quality was increased. This difference suggests a disparity in beliefs about meat quality. While meat quality is generally described in the West using attributes such as aesthetic, taste and nutritional value, in halal slaughter there is also an underlying dimension of spirituality [34]. Ensuring that religious requirements are met may supersede any other properties of the meat [34] and in a spiritual way, the quality of the meat is improved from the perspective of Muslims.

A commonly-held belief among participants in both countries was that stunning of animals is not allowed in halal slaughter, with 52% stating that animals can never be stunned in halal slaughter. In Australia most halal-approved abattoirs use reversible stunning during halal slaughter [10], with very few abattoirs permitted to slaughter animals without the use of stunning for halal and kosher purposes [10]. It is unknown how this belief originated, but it is possible participants in Australia had been influenced by the media commenting on these few exceptions.

In Malaysia some nonlethal methods of stunning have been adopted by Muslim scholars provided certain pre-requisites are fulfilled [35]. This is in line with Halal Malaysia certification guidelines which state that to render the animal immobile or unconscious, various methods of stunning that allow the animal to bleed out while it is still alive can be utilised [4]. These methods of stunning are subject to approval by the Malaysian Fatwa Council degree and in particular, must be reversible and not lead to death or cause permanent physical injury to the animal [4]. It is unknown whether Malaysian participants who believed that stunning was never allowed in halal slaughter had only been exposed to slaughter without the use of stunning, or whether they were simply unaware of stunning being practiced at abattoirs.

### Halal acceptance

Participants were more likely to have a positive attitude to halal slaughter if they had a higher level of education, and were also more likely to believe that it was important to provide halal options within Malaysian or Australian societies. This supports the claim that better educated people tend to have more favorable attitudes to immigration and/or diversity [36].

Muslims were more likely than those of other religious groups to consider the humane and respectful treatment of animals in halal to be important. The Qu’ran does not sanction mistreatment of animals [37, 6]. As Islam teaches that animals should be slaughtered in a mindful and attentive way, scientific wisdom now leads many to accept that animals must first be stunned prior to slaughter in order to avoid compromising their welfare [34]. In Morality Without Religion, Peter Singer & Marc Hauser discussed the concept that “What was good for our ancestors may not be good for human beings as a whole today, let alone for our planet and all the other beings living on it.” [38] Under this notion, the respect afforded to animals in the Qu’ran could be applied using current day knowledge to minimise suffering. Singer has also suggested that the traditional link between religion and ethics is that religion provides “a reason for doing what is right”. [39]

The Sunnah states that water should be provided to animals prior to slaughter and a very sharp knife must be used in order to swiftly slaughter the animal causing minimum suffering. It discourages slaughtering animals in plain view of other animals, skinning or cutting animals prior to death. Non-Muslims may be less aware of the importance of these matters during halal slaughter. This supports the findings of Ayyub [40] that non-Muslims perceived cruelty in Halal and believed that the Halal slaughter process causes pain and suffering, but were unaware of the actual difference between Halal and non-Halal products. British respondents have also referenced Youtube videos and other documentary media sources as part of their knowledge on Halal [40], which has similarities to the Australian public developing concerns about some Halal slaughter following media coverage of practices in Indonesia [8].

Even within Malaysia, non-Muslims have been previously shown to have a low understanding of fundamental halal principles [41]. with only 40% of respondents found to properly understand the halal principles and the majority not believing that the halal concept is “fully concerned with animal welfare”.

There were some limitations of the study. As considered at the start of this Discussion, the respondents may not have been representative of Malaysian and Australian populations, but to guard against this possibility respondents were obtained from multiple locations in each country. The difference in the most commonly identified living place, suburban for Australians and inner city for Malaysians, may have been because ‘suburban’ cannot be easily translated into Malay language. However, place of residence did not have significant effects on responses, so this was not considered to have biased results.

## Conclusions

This study provides a preliminary understanding of attitudes towards halal slaughter in an influential Islamic country, Malaysia, and a Western country with a multicultural society, Australia. This information could be beneficial when addressing the needs of society for provision of meat that is acceptable according to the religious scriptures, whilst at the same time meeting the needs of society and the relevant religions in regards to the welfare of livestock during slaughter. The important role of education was demonstrated, and the lack of certainty about which practices are required for halal slaughter, particularly in Australia, emphasises that there is a role for targeted education in this field.

Recognition that stunning could be used within halal slaughter was limited, and again education of the suitability of a reversible stun to accompany halal slaughter could be beneficial. This education could commence at school level, as it has been previously demonstrated children can develop their understanding of Halal concepts at an early age, with comments from a predominantly Muslim group of children that Halal meat was “healthy” and “good.” [42]. A basic explanation of how animals can be stunned to provide better welfare in Halal slaughter could be introduced both to Muslim and non-Muslim children to encourage acceptance of the practice.

Increasing knowledge of what is and is not permissible under halal slaughter could alter attitudes with regard to halal animal products among both non-Muslims and Muslims, who are demonstrated to be unclear about some of the stipulations that their religious tradition requires. Women and older people were more concerned than men or younger participants and educational campaigns should probably focus on these groups if they are perceived to be potential arbiters of change. Women are more likely to be purchasers than men, and the views of older people is likely to be more respected than that of younger people, hence they may well be suitable to engender change.

Further studies could consider a more detailed analysis of what will motivate other consumers to purchase halal products with higher animal welfare standards. A future study could also be considered into Jewish awareness of the processes of Kosher slaughter, with the Jews surveyed attributing the least importance value to the humane and respectful treatment of animals in halal slaughter and finding the slaughter of conscious animals for religious reasons unacceptable. The general acceptance that slaughter should be legally controlled suggests that the Australian and Malaysian governments would receive public support in developing strict standards for animal welfare during slaughter. Qualitative studies into what both Muslims and non-Muslims perceive to be good animal welfare in relation to halal slaughter practices, in order to avoid states such as pain, fear, and distress in animals would potentially draw out key underlying themes that have not emerged in this analysis.

## Supporting information

S1 File Halal data. (XLSX)
